# Hypoxia ameliorates neurodegeneration and movement disorder in a mouse model of Parkinson’s disease

**DOI:** 10.1038/s41593-025-02010-4

**Published:** 2025-08-06

**Authors:** Eizo Marutani, Maria Miranda, Timothy J. Durham, Sharon H. Kim, Dreson L. Russell, Presli P. Wiesenthal, Paul Lichtenegger, Marissa A. Menard, Charlotte F. Brzozowski, Haobo Li, Gary Ruvkun, Joshua D. Meisel, Laura Volpicelli-Daley, Vamsi K. Mootha, Fumito Ichinose

**Affiliations:** 1 Anesthesia Center for Critical Care Research of the Department of Anesthesia Critical Care and Pain Medicine, Massachusetts General Hospital, Boston, MA USA; 2 Harvard Medical School, Boston, MA USA; 3 Howard Hughes Medical Institute and Department of Molecular Biology, Massachusetts General Hospital, Boston, MA USA; 4 Department of Systems Biology, Harvard Medical School, Boston, MA USA; 5 Broad Institute of MIT and Harvard, Cambridge, MA USA; 6 Center for Neurodegeneration and Experimental Therapeutics, Department of Neurology, University of Alabama at Birmingham, Birmingham, AL USA; 7 Department of Molecular Biology, Massachusetts General Hospital, Boston, MA USA; 8 Department of Anesthesia Critical Care and Pain Medicine, Massachusetts General Hospital, Boston, MA USA; 9 Present Address: Department of Biology, Brandeis University, Waltham, MA USA

**Keywords:** Parkinson's disease, Parkinson's disease, Cell death in the nervous system

## Abstract

Parkinson’s disease (PD) is characterized by inclusions of α-synuclein (α-syn) and mitochondrial dysfunction in dopaminergic (DA) neurons of the substantia nigra pars compacta (SNpc). Patients with PD anecdotally experience symptom improvement at high altitude; chronic hypoxia prevents the development of Leigh-like brain disease in mice with mitochondrial complex I deficiency. Here we report that intrastriatal injection of α-syn preformed fibrils (PFFs) in mice resulted in neurodegeneration and movement disorder, which were prevented by continuous exposure to 11% oxygen. Specifically, PFF-induced α-syn aggregation resulted in brain tissue hyperoxia, lipid peroxidation and DA neurodegeneration in the SNpc of mice breathing 21% oxygen, but not in those breathing 11% oxygen. This neuroprotective effect of hypoxia was also observed in *Caenorhabditis elegans*. Moreover, initiating hypoxia 6 weeks after PFF injection reversed motor dysfunction and halted further DA neurodegeneration. These results suggest that hypoxia may have neuroprotective effects downstream of α-syn aggregation in PD, even after symptom onset and neuropathological changes.

## Abstract (web summary)

Parkinson’s disease (PD) involves toxic protein buildup and energy failure in neurons. Continuously breathing low-oxygen air protected mice from PD-like neuronal loss and reversed symptoms, even after they began, suggesting that hypoxia may protect neurons.

## Main

Parkinson’s disease (PD) is a chronic and progressive multisystem neurodegenerative disease that affects millions of individuals. The cardinal motor symptoms of PD include tremor, rigidity, bradykinesia and akinesia, and postural instability. However, the clinical presentation includes a variety of motor and non-motor symptoms^1^. Pathologically, PD is characterized by the selective loss of midbrain dopaminergic (DA) neurons in the substantia nigra pars compacta (SNpc) and the accumulation of intracellular inclusions called Lewy bodies (LBs) and Lewy neurites (LNs), which mainly consist of aggregated fibrillar α-synuclein (α-syn)^2^. α-Syn is a presynaptic protein consisting of 140 amino acids and encoded by *SNCA*. While the function of α-syn is not well understood, α-syn fibrils formed during the early stages of aggregation are considered highly toxic to many intracellular processes and organelles, notably mitochondria^3,4^.

Mitochondrial dysfunction has long been implicated in PD^5^. Pathological α-syn can inhibit mitochondrial complex I (MCI), leading to mitochondrial fragmentation, reduced membrane potential, impaired axonal transport, decreased mitochondrial gene expression, disrupted oxidative phosphorylation and increased reactive oxygen species (ROS) production^6–12^. Chemical inhibitors of MCI, notably the pesticide rotenone and 1-methyl-4-phenyl-1,2,3,6-tetrahydropyridine (a product of meperidine analog synthesis), are toxic to DA neurons and can induce parkinsonism^13,14^. In addition, biochemical MCI deficiencies occur in the SNpc of cases with sporadic PD^5^, and many recessive forms of familial PD affect mitochondrial proteins^15,16^. Genetic ablation of MCI in DA neurons is sufficient to cause parkinsonism in mice^17^. Collectively, these observations underscore the critical role of mitochondrial dysfunction in the pathogenesis of both sporadic and familial forms of PD. Unfortunately, no therapies currently exist to halt or slow the progression of neurodegeneration in patients with PD.

Monogenic defects in MCI are a common biochemical cause of Leigh syndrome, the most prevalent pediatric manifestation of mitochondrial disease characterized by neurodegeneration affecting the deep gray matter^18^. Mice deficient in *Ndufs4*, which encodes the MCI subunit NADH:ubiquinone oxidoreductase subunit S4, have decreased MCI activity and serve as a model of Leigh syndrome by recapitulating many of the neuropathological and clinical features^19^. Previous research demonstrated that continuous exposure to 11% O_2_ prevents neurodegeneration and markedly extends the lifespan (approximately fivefold) in *Ndufs4*^−/−^ mice^20^. When administered to mice with advanced disease, it can halt and even reverse pathology^21^. More recent studies demonstrated that hypoxia rescue of MCI deficiency is evolutionarily conserved in *Caenorhabditis elegans*^22^. Moreover, patients with PD anecdotally experience improvement in their symptoms at high altitude, where inspired oxygen levels are reduced^23^. Based on these observations, we hypothesized that hypoxia treatment might prevent degeneration of DA neurons in the SNpc and alleviate movement disorder in PD.

## Results

### Hypoxia prevents α-syn-induced loss of DA neurons in SNpc

**Fig. 1:**
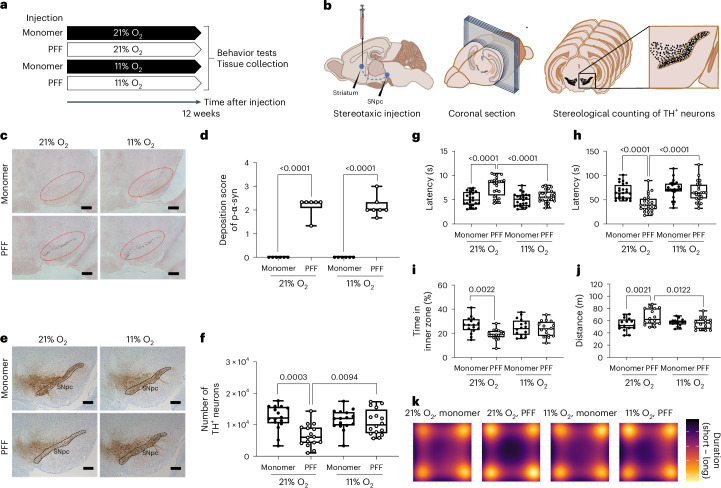
Hypoxia treatment prevents α-syn aggregate-induced loss of DA neurons in the SNpc and movement disorder. **a**, Schematic depicting the experimental groups and timeline. **b**, Schematic depicting stereological cell counting in the SNpc. The total number of TH^+^ neurons in the entire SNpc was counted at an average of six sections per animal. **c**, Representative photomicrograph of SNpc immunostained for phosphorylated α-syn in mice treated with PFF or α-syn monomer and exposed to 21% or 11% O_2_ for 12 weeks, with a schematic indicating the location of the SNpc captured. **d**, Deposition score of phosphorylated α-syn in the SNpc of mice treated with PFF or α-syn monomer and exposed to 21% or 11% O_2_ for 12 weeks. **c**,**d**, Number of mice in each group: 21% O_2_, monomer (*n* = 6); 21% O_2_, PFF (*n* = 6); 11% O_2_, monomer (*n* = 6); and 11% O_2_, PFF (*n* = 7). A two-way analysis of variance (ANOVA) followed by Tukey’s correction for post-hoc comparisons was conducted. **e**, Representative photomicrograph of SNpc immunostained for TH in mice treated with PFF or α-syn monomer and exposed to 21% or 11% O_2_ for 12 weeks. **f**, Number of stereologically counted TH^+^ neurons in the SNpc of mice treated with PFF or α-syn monomer and exposed to 21% or 11% O_2_ for 12 weeks (*n* = 16 mice in each group). A two-way ANOVA followed by Tukey’s correction for post-hoc comparisons was conducted. **g**–**j**, Results of the pole (**g**) and cage hang (**h**) tests, time spent in the inner zone (**i**) and total distance (**j**) in the open field test in mice treated with PFFs or α-syn monomer and exposed to 21% or 11% O_2_ for 12 weeks (*n* = 24 in each group in **g** and **h**). **i**,**j**, *n* = 16 in each group. **g**–**j**, A two-way ANOVA, followed by Šídák’s correction for post-hoc comparisons, was conducted. **k**, Heatmap showing the location of mice during the open field test. Each image shows the average latency of mice in each group. **d**,**f**–**j**, Data are presented as box plots showing the median value and interquartile range (IQR), with the whiskers denoting the maximum and minimum values. **c**,**e**, Scale bars, 100 μm. Source data

We investigated whether chronic exposure to moderate environmental hypoxia, that is, continuously breathing 11% O_2_ at sea level, could ameliorate neurodegeneration and motor dysfunction in mice subjected to intrastriatal injection of α-syn preformed fibrils (PFFs). A fraction of inspired oxygen (FiO_2_) of 11% at sea level corresponds to an inspired partial pressure of O_2_ of 78.43 mmHg, which is equivalent to breathing 21% O_2_ at an altitude of 4,500–4,999 m^24^. We recently reported that chronic, continuous breathing of 11% O_2_ is well tolerated by healthy volunteers after several days of stepwise acclimatization in a hospital setting^25^. Injection of α-syn PFFs induces endogenous α-syn to form inclusions that biochemically and morphologically resemble those found in PD^4,26^. After receiving a unilateral injection of PFFs or monomeric α-syn as control into the right striatum, mice were housed in custom-made chambers where they continuously breathed either normoxic (21% O_2_) or hypoxic (11% O_2_) gas at normobaria for 12 weeks (Fig. 1a). To examine the effects of hypoxia treatment on PFF-induced α-syn aggregation and the integrity of DA neurons, we immunolabeled α-syn phosphorylated at Ser129, a marker of human LBs and LNs, and performed unbiased stereological counting of tyrosine hydroxylase (TH)^+^ DA neurons in coronal brain sections containing the SNpc of brain obtained 12 weeks after intrastriatal injection of PFF or α-syn monomer (Fig. 1b)^27^. In mice breathing 21% O_2_, PFFs induced the formation of dense LB-like inclusions (Fig. 1c,d) and the loss of TH^+^ DA neurons (Fig. 1e,f) in the ipsilateral SNpc by 12 weeks. In mice breathing 11% O_2_, PFF-injected individuals showed a similar degree of phosphorylated α-syn deposition as the PFF-injected mice breathing 21% O_2_ (Fig. 1c,d). However, breathing 11% O_2_ rescued the loss of TH^+^ DA neurons in the SNpc of PFF-treated mice (Fig. 1e,f). These results indicate that hypoxia treatment in mice with PFF-induced α-syn inclusion formation mitigates the loss of TH^+^ DA neurons, without preventing the formation of Lewy pathology-like inclusions in the SNpc.

### Hypoxia prevents α-syn-induced parkinsonism in mice

**Extended Data Fig. 1:**
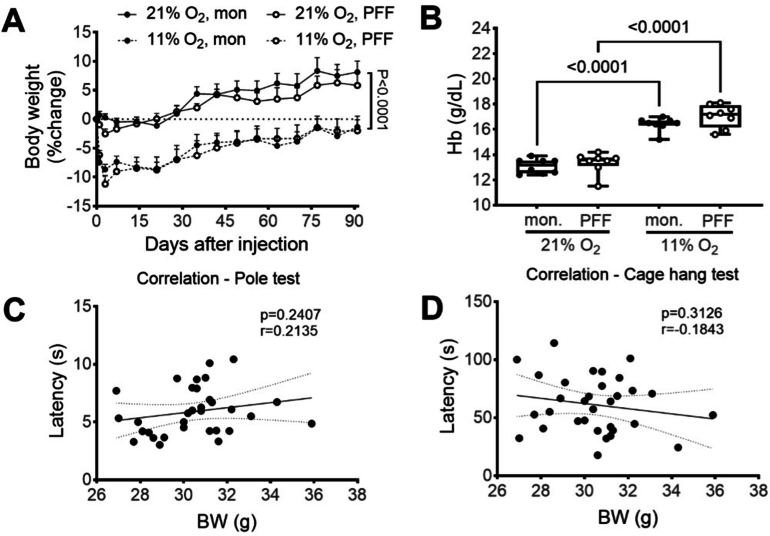
Impact of PFF injection and hypoxia treatment on body weight, hemoglobin concentration and performance of behavior tests. Changes in body weight (**a**) and hemoglobin concentration (**b**) in mice breathing 21% or 11% O_2_ for 12 weeks after injection of monomer or PFF. N = 8 in each group. Two-way ANOVA followed by Tukey’s correction for post-hoc comparisons was conducted for panel (**a**) and (**b**). The Pearson correlation coefficient (r) with two-tailed hypothesis was calculated for panel (**c**) and (**d**). N = 32. Source data

Motor function and behavior were assessed using the pole, cage hang and open field tests 12 weeks after injection^26,28^. The pole test models motor coordination and bradykinesia. The cage hang test (also known as the wire hang test) measures muscle strength and endurance^29,30^. The open field test (OFT) assesses anxiety-like behavior. When breathing 21% O_2_, compared to monomeric α-syn-treated controls, mice with PFF-induced α-syn aggregates exhibited prolonged time to climb down in the pole test (Fig. 1g) and reduced latency to hang onto the wire in the cage hang test (Fig. 1h). Moreover, they exhibited increased anxiety-like behavior in the OFT as evidenced by decreased time spent in the inner zone (Fig. 1i) and increased total distance traveled (Fig. 1j) compared to controls. In contrast, when breathing 11% O_2_, PFF-injected mice performed similarly to control mice on all three tests. In monomeric α-syn-treated mice, breathing 11% O_2_ did not affect latency in the pole and cage hang tests, nor did it affect the time spent in the inner zone or the total distance moved in the OFT compared to breathing 21% O_2_ (Fig. 1k). Although breathing 11% O_2_ caused a transient decrease in body weight of approximately 10% in all mice for the first few days, it did not affect the rate of body weight gain thereafter (Extended Data Fig. 1a). PFF-treated and α-syn-monomer-treated mice exhibited similar increase in hemoglobin concentration in response to hypoxia (Extended Data Fig. 1b). There was no correlation between body weight and the results of the pole and cage hang tests (Extended Data Fig. 1c,d). These results suggest that continuous exposure to 11% O_2_ prevents the development of movement disorder and anxiety-like behavior in mice with α-syn aggregates.

### Hypoxia prevents hyperoxia and lipid peroxidation in SNpc

**Fig. 2:**
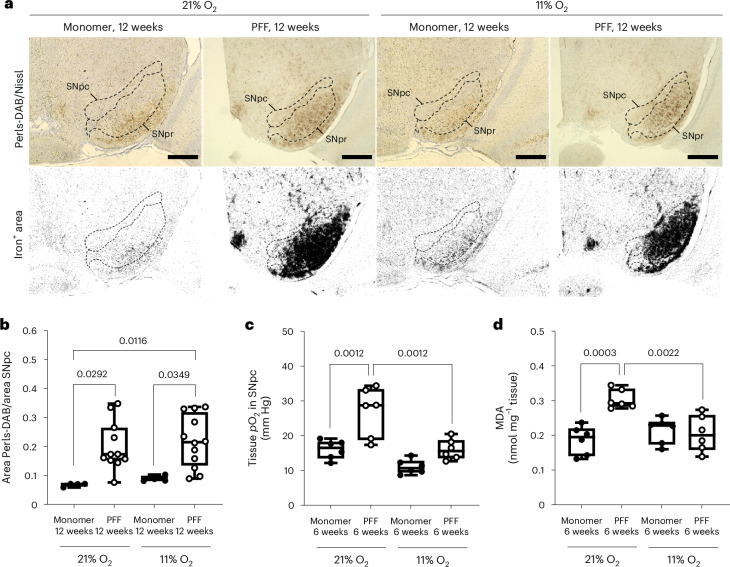
Hypoxia prevents PFF-induced tissue hyperoxia and lipid peroxidation in the SNpc of mice. **a**, Representative photomicrograph of Perls-DAB-stained SNpc in mice treated with PFF or α-syn monomer and breathing 21% or 11% O_2_ for 12 weeks. **b**, Ratio of Perls-DAB^+^ area to SNpc area in mice treated with PFF or α-syn monomer and breathing 21% or 11% O_2_ for 12 weeks. **a**,**b**, *n* = 4 for mice injected with α-syn monomer; *n* = 11 or 12 for mice injected with PFF. **c**, Brain tissue *p*O_2_ in the SNpc of mice treated with PFF or α-syn monomer and breathing 21% or 11% O_2_ for 6 weeks. *n* = 6 in each group. **d**, MDA levels in the SN of mice treated with PFF or α-syn monomer and breathing 21% or 11% O_2_ for 6 weeks. *n* = 6 in each group. A two-way ANOVA followed by Tukey’s correction for post-hoc comparisons was conducted. **b**–**d**, Data are presented as box plots showing the median and IQR, with the whiskers denoting the maximum and minimum values. **a**, Scale bars, 100 μm. SNpr, substantia nigra pars reticulata. Source data

Neuronal iron accumulation in the substantia nigra (SN) is a hallmark of PD pathology^31^. To determine whether hypoxia affects iron metabolism in the SN after α-syn aggregation, we examined iron levels in the SNpc. The area of the SNpc was delineated based on anatomical landmarks using a mouse brain atlas by an investigator blinded to the identity of the samples. Iron accumulation, as measured using Perls-3,3′-diaminobenzidine (DAB) staining, increased in the SNpc at 12 weeks after PFF injection, compared to α-syn monomer injection (Fig. 2a,b). Hypoxia had no effect on iron levels in the SNpc of both α-syn-monomer-injected and PFF-injected mice, indicating that the beneficial effects of hypoxia after PFF injection are not mediated by attenuation of iron accumulation.

Because prior studies showed mitochondrial respiratory chain dysfunction induced by α-syn^6–8^, and because primary mitochondrial dysfunction can result in tissue hyperoxia^32^, we wondered if the partial pressure of oxygen (*p*O_2_) is elevated in the SNpc of our PD mouse model. To our knowledge, no prior study measured brain *p*O_2_ in patients with PD or in mouse models of α-syn toxicity. We measured the *p*O_2_ in the SNpc using a fiber-optic fluorescence oxygen sensor 6 weeks after intrastriatal injection of PFF or monomer. When breathing 21% O_2_, mice injected with PFF exhibited higher brain tissue *p*O_2_ in the SNpc compared to mice injected with α-syn monomer (Fig. 2c). In contrast, breathing 11% O_2_ inhibited the PFF-induced increase in brain tissue *p*O_2_ in the SNpc. There could be multiple mechanistic explanations for the observed relative hyperoxia, including decreased oxygen consumption by the mitochondrial respiratory chain due to PFF inhibiting MCI^6–8^.

We hypothesized that local hyperoxia in the PFF-injected SNpc of mice breathing 21% O_2_ could promote lipid peroxidation. Recent genome-wide CRISPR genetic screens revealed a strong synthetic lethal interaction between mitochondrial respiratory chain inhibition and genetic loss of GPX4, a lipid hydroperoxidase^33^. Moreover, oncocytic thyroid carcinoma is associated with somatic genetic loss of MCI and is highly sensitive to death from lipid peroxidation^34^. To test this hypothesis, we measured malondialdehyde (MDA) levels in the SN 6 weeks after intrastriatal injection of α-syn PFF or monomer. In mice breathing 21% O_2_, those with PFF-induced α-syn aggregates exhibited higher MDA levels in the SN compared to monomer-injected mice (Fig. 2d). In contrast, breathing 11% O_2_ attenuated the PFF-induced increase in MDA levels in the SN. These observations suggest that hypoxia attenuates lipid peroxidation in the SN after α-syn aggregation by mitigating brain tissue hyperoxia without affecting iron accumulation.

### Hypoxia alters transcriptional response to α-syn in the SN

**Fig. 3:**
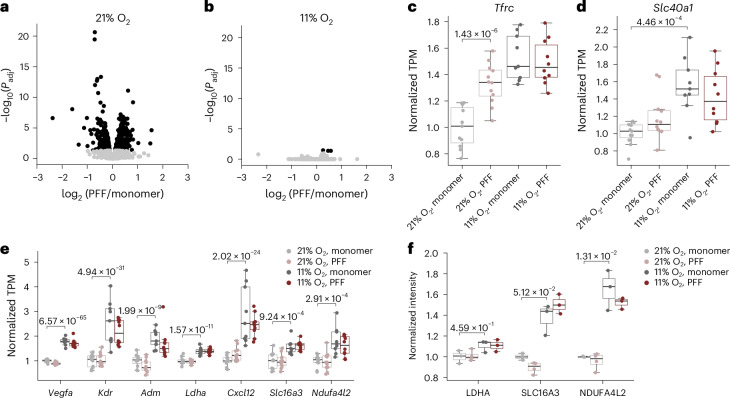
PFF-induced α-syn aggregates result in dramatic transcriptomic changes in the SN in mice breathing 21% O_2_, but not in mice breathing 11% O_2_. **a**, Volcano plot showing the log_2_ fold change of transcripts in the SN 12 weeks after striatal injection of PFF versus α-syn monomer in mice breathing 21% O_2_, plotted against the −log_10_(*P*_adj_). Significantly changing genes (log_2_fold change > 0.25, *P*_adj_ < 0.05 from a DESeq2 Wald test with multiple testing using the Benjamini–Hochberg procedure) are denoted in black. **b**, Volcano plot showing the log_2_fold change of transcripts in the SN 12 weeks after striatal injection of PFF versus α-syn monomer in mice breathing 11% O_2_, plotted against the −log_10_(*P*_adj_). Significantly changing genes (log_2_fold change > 0.25, *P*_adj_ < 0.05) are denoted in black. **c**,**d**, Box plot showing *Tfrc* (**c**) and *Slc40a1* (*Fpn*) (**d**) expression. Significance is indicated using *P*_adj_ calculated in the DESeq2 analysis of all genes (that is, DESeq2 Wald test with multiple testing correction using the Benjamini–Hochberg procedure). **e**, Box plot showing HIF targets upregulated in the SN of mice breathing 11% O_2_. The *y* axes in the box plots represent the transcripts per million (TPM) normalized for each gene to the mean TPM of α-syn-monomer-treated mice breathing 21% O_2_. The box plots are annotated with the adjusted *P* values calculated in the DESeq2 analysis of all genes (that is, DESeq2 Wald test with multiple testing correction using the Benjamini–Hochberg procedure). **a**–**e**, The number of mice in each group was 21% O_2_, monomer (*n* = 11); 21% O_2_, PFF (*n* = 12); 11% O_2_, monomer (*n* = 9); and 11% O_2_, PFF (*n* = 10). **f**, Box plot showing HIF target proteins upregulated in the SN of mice breathing 11% O_2_. The *y* axes in the box plots represent the TMT intensity normalized for each gene to the mean intensity of α-syn-monomer-treated mice breathing 21% O_2_. The number of mice in each group was 21% O_2_, monomer (*n* = 3); 21% O_2_, PFF (*n* = 4); 11% O_2_, monomer (*n* = 3); and 11% O_2_, PFF (*n* = 3). Box plots are annotated with the adjusted *P* values calculated in the differential expression analysis of all proteins using limma (that is, a two-tailed moderated *t*-test with multiple testing correction using the Benjamini–Hochberg procedure). For all box plots, the center line shows the median, the box shows the IQR and the whiskers show the extent of the data distribution up to 1.5 times the IQR. Source data

**Extended Data Fig. 2:**
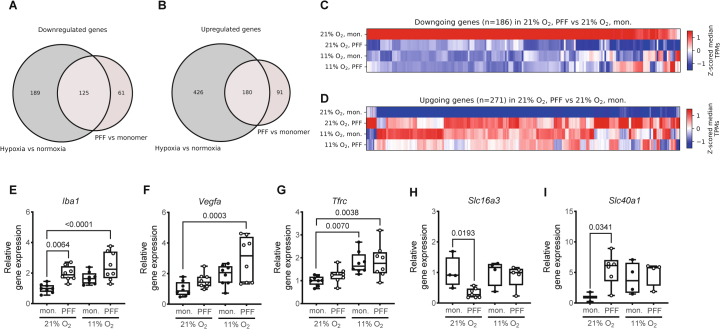
RNA-seq, TMT proteomics, qPCR. (**a**) and (**b**): Venn diagrams show the numbers of shared and distinct downregulated (**a**) and upregulated (**b**) genes when comparing the transcriptomic effects of hypoxia treatment (11% O_2_, monomer vs 21% O_2_, monomer) to the effects of PFF treatment (21% O_2_, PFF vs 21% O_2_, monomer). (**c**) and (**d**): Heatmaps show the z-score scaled expression across condition for all 186 downregulated genes (**c**) and all 271 upregulated genes (**d**) in the 21% O_2_, PFF versus 21% O_2_, monomer comparison. Columns (genes) were hierarchically clustered using fastcluster.linkage (v1.2.6), with metric=euclidean and method=single. N = 12 in each group. Results of RT-qPCR for *Iba1* (**e**), *Vegfa* (**f**), *Tfrc* (**g**), *Slc16a3* (**h**) and *Slc40a1* (**i**). N = 3-8. Two-way ANOVA followed by Dunnett’s correction for post-hoc comparisons was conducted for panel (**e**–**i**). Source data

To better understand how hypoxia confers neuroprotection against α-syn aggregates, we performed RNA sequencing (RNA-seq) of the SN (Methods). We observed that α-syn aggregation induced remarkable changes in the transcriptome of mice breathing 21% O_2_, as evidenced by a striking eruption in the volcano plot (Fig. 3a). However, it elicited no such transcriptional eruption in mice breathing 11% O_2_ (Fig. 3b). In fact, the transcriptional changes induced by α-syn aggregates in mice breathing 21% O_2_ were very similar to the changes in control mice (treated with α-syn monomer) breathing 11% O_2_ compared to α-syn monomer treatment in mice breathing 21% O_2_ (Extended Data Fig. 2a–d). We conclude that PFF-induced α-syn aggregation triggers toxicity and is associated with many transcriptional changes, but that hypoxia gives rise to a cellular state such that PFF aggregates have little additional differential impact.

Among the genes most upregulated by PFF-induced α-syn aggregation in mice breathing 21% O_2_, and also increased by breathing 11% O_2_, is *Tfrc* (Fig. 3c), which encodes TFR1 and is central to the endocytic import of transferrin-bound iron. We also observed a significant increase of *Slc40a1* (ferroportin, *Fpn*), the main iron transporter, by breathing 11% O_2_ in the SN (Fig. 3d). Neuronal iron accumulation in the SNpc is a hallmark of PD pathology^31^ and is recapitulated in our model (Fig. 2a,b). A recent study using the same mouse model of PD reported that after PFF exposure, activated microglia secrete interleukin-6, which leads to transcriptional upregulation of *Tfrc* in neurons and contributes to neuronal iron accumulation^35^. Although our bulk RNA-seq of the SN of mice with intrastriatal injection of PFF does not show an interleukin-6 signature, we observed a marked increase of *Iba1* mRNA levels by quantitative PCR with reverse transcription (RT–qPCR) in the SN of an independent cohort of mice 3 months after PFF injection (Extended Data Fig. 2e). Upregulation of *Tfrc* and *Fpn* by hypoxia and or α-syn PFF observed in the RNA-seq was also corroborated using RT–qPCR in the independent cohort of mice (Extended Data Fig. 2g,i). These observations are consistent with the role of microglial activation in iron accumulation in PD, and the profile of iron accumulation in the SN observed in our study (Fig. 2a,b).

A number of hypoxia-inducible factor (HIF) targets are upregulated in response to hypoxia, some of which may be important in conferring neuroprotection. Canonical HIF targets, including *Vegfa*, *Kdr*, *Adm*, *Ldha*, *Cxcl12* and *Slc16a3* are upregulated with hypoxia (Fig. 3e); we corroborated many of these further using either RT–PCR (Extended Data Fig. 2e–i) or tandem mass tag (TMT) proteomics (Fig. 3f) in independent cohorts of mice (Methods). *Ldha*, which encodes lactate dehydrogenase, elevated at the RNA level and trending up at the protein level, and *Slc16a3*, which encodes the *Mct4* monocarboxylate transporter, elevated at the RNA and protein level, are both particularly interesting because recent studies have shown that the HIF-dependent lactate axis drives resistance against ferroptosis in the context of cancer^36^. Another notable known HIF target is *Ndufa4l2*, which is upregulated by hypoxia both at the RNA and protein levels. It encodes a component of the electron transport chain that dampens its activity as a protective mechanism^37,38^, which may be particularly relevant in the setting of PFF toxicity as it is known to affect mitochondria.

### Hypoxia remains beneficial at 10 months after PFF injection

**Fig. 4:**
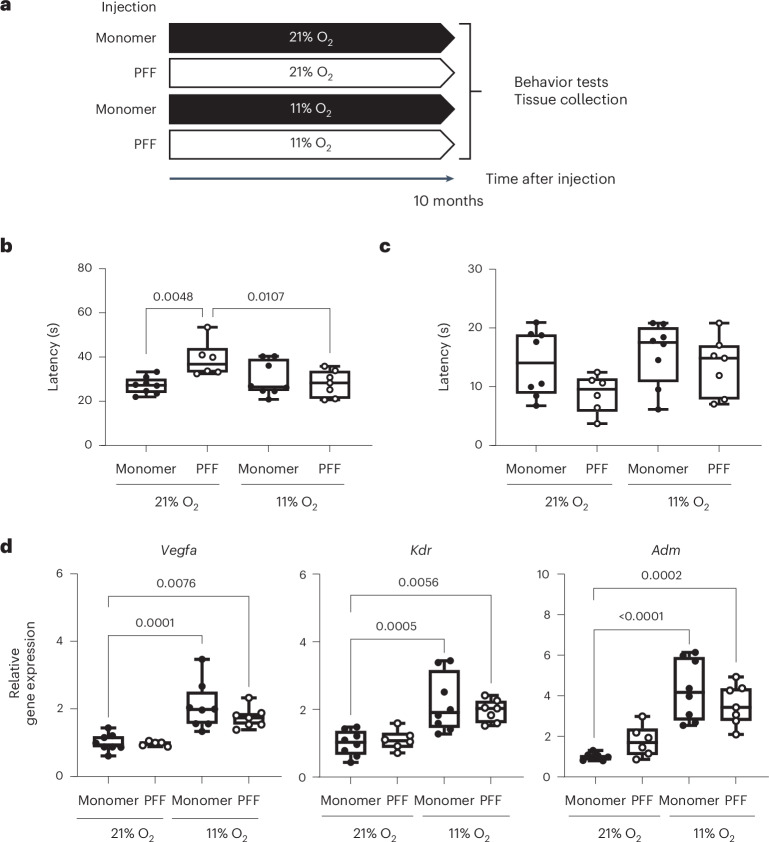
Breathing 11% O_2_ remains beneficial 10 months after PFF-induced α-syn aggregation. **a**, Schematic depicting the experimental groups and timeline. **b**,**c**, Results of the pole (**b**) and cage hang (**c**) tests in mice treated with PFF or α-syn monomer and exposed to 21% or 11% O_2_ for 10 months. **b**,**c**, The number of mice in each group was 21% O_2_, monomer (*n* = 8); 21% O_2_, PFF (*n* = 6); 11% O_2_, monomer (*n* = 8); and 11% O_2_, PFF (*n* = 7). **d**, Relative gene expression levels of *Vegfa*, *Kdr* and *Adm* in the SN of mice treated with PFF or α-syn monomer and exposed to 21% or 11% O_2_ for 10 months. The number of mice in each group was 21% O_2_, monomer (*n* = 8); 21% O_2_, PFF (*n* = 5 for *Vegfa*, *n* = 6 for *Kdr* and *Adm*); 11% O_2_, monomer (*n* = 8); and 11% O_2_, PFF (*n* = 7). **b**–**d**, A two-way ANOVA followed by Dunnett’s correction for post-hoc comparisons was conducted. Data are presented as box plots showing the median value and IQR, with the whiskers denoting the maximum and minimum values. Source data

**Extended Data Fig. 3:**
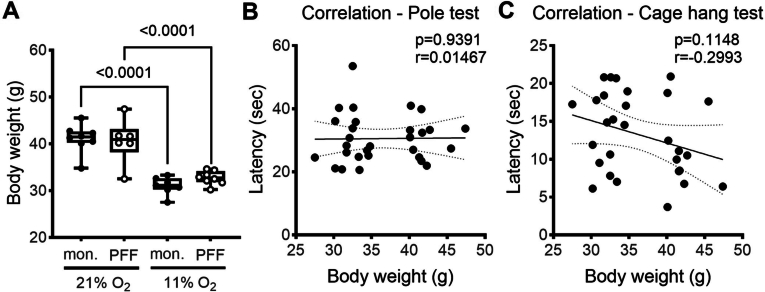
Impact of PFF injection and hypoxia treatment on body weight and performance of behavior tests. Changes in body weight (**a**) in mice breathing 21% or 11% O_2_ for 10 months after injection of monomer or PFF. N = 6-8 in each group. Two-way ANOVA followed by Tukey’s correction for post-hoc comparisons was conducted for panel (**a**). The Pearson correlation coefficient (r) with two-tailed hypothesis was calculated for panel (**b**) and (**c**). N = 29. Source data

To determine the long-term effects of breathing 11% O_2_ in the PD model, motor function was examined using the pole and cage hang tests in a cohort of mice that breathed 21% or 11% O_2_ for 10 months after injection of PFF or α-syn monomer (Fig. 4a). Continuously breathing 11% O_2_ for 10 months after injection of α-syn PFF was well tolerated and remained beneficial; breathing 11% O_2_ for 10 months prevented PFF-induced prolongation of latency in the pole test and tended to prevent PFF-induced shortening of latency in the cage hang test (Fig. 4b,c). While body weight was lighter in mice breathing hypoxia than mice breathing normoxia, there was no correlation between the body weight of mice and latency of either test (Extended Data Fig. 3a–c). HIF targets involved in vasculogenesis, including *Vegfa*, *Kdr* and *Adm*, remained upregulated in the SN after breathing 11% O_2_ for 10 months, suggesting robust impact of HIF-dependent signaling after chronic hypoxia breathing (Fig. 4d). These observations support the protective effects of continuously breathing 11% O_2_ at least up to 10 months after α-syn aggregation.

### Hypoxia prevents loss of DA neurons in *Caenorhabditis elegans*

**Fig. 5:**
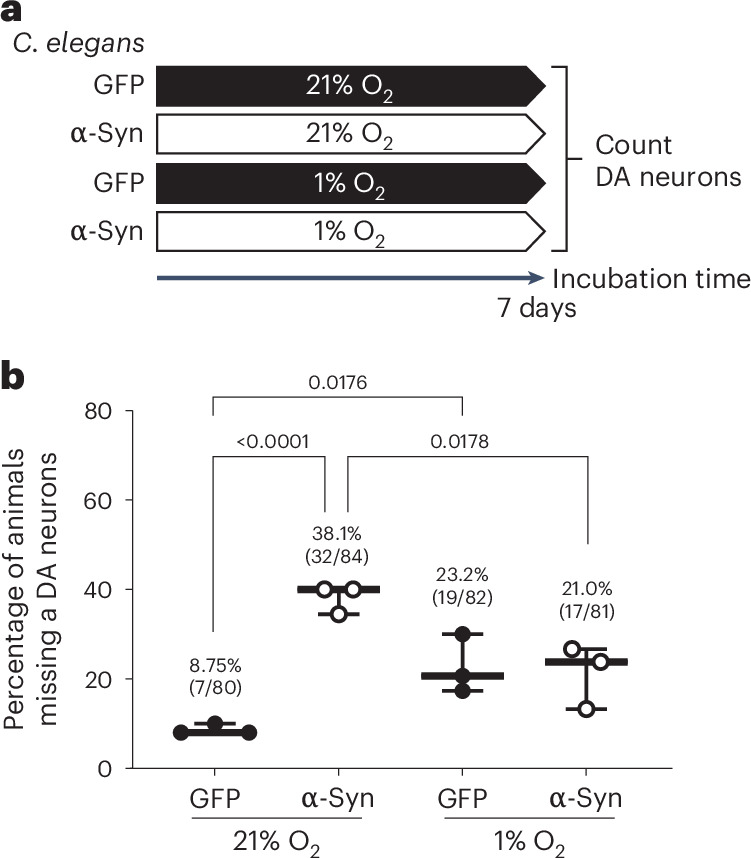
Hypoxia prevents the loss of DA neurons in *C. elegans* overexpressing α-syn. **a**, Schematic depicting the experimental groups and timeline. **b**, Percentage of *C. elegans* in which at least one DA cephalic (CEP) neuron is absent at day 7 of adulthood. Each point represents one cohort in which approximately 30 animals were analyzed (the number of *C. elegans* in each group was 21% O_2_, GFP (*n* = 80); 21% O_2_, α-syn (*n* = 84); 1% O_2_, GFP (*n* = 82); and 1% O_2_, α-syn (*n* = 81)). **b**, Data are presented as a box plot showing the median and IQR, with the whiskers denoting the maximum and minimum values. A Fisher’s exact test was conducted to compare each of two groups with a two-sided *P* value. Source data

**Extended Data Fig. 4:**
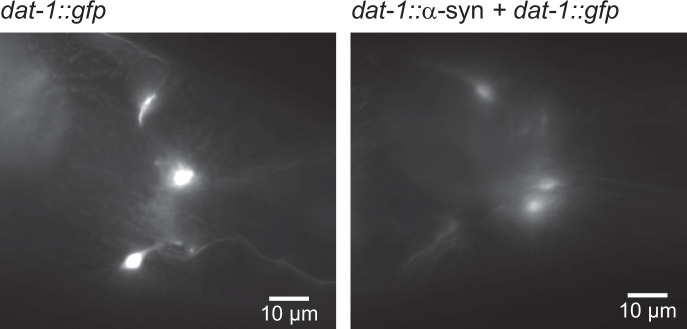
Representative images of *C. elegans* expressing GFP alone or α-syn and GFP. *C. elegans* normally have four DA CEP neurons in the head. In animals grown in 21% O_2_, four DA CEP neurons are present in the wild-type animal (*dat-1::gfp*) and three visible in this focal plane. Only three DA neurons are present in the transgenic animal expressing *α*-syn (*dat-1::α-syn + dat-1::gfp*). Lower GFP fluorescence in the *α*-syn-expressing animal is indicative of neurons in the process of degeneration.

To determine if hypoxia rescue of α-syn-induced neurodegeneration is evolutionarily conserved, we analyzed a well-established *C. elegans* system in which overexpression of human α-syn causes age-dependent loss of DA neurons^39,40^. We compared strains expressing green fluorescent protein (GFP) alone (wild-type (WT)) or GFP and α-syn in DA neurons and grew animals continuously in either 21% or 1% O_2_, a condition previously shown to rescue certain forms of complex I dysfunction in *C. elegans*^22^. In animals grown in 21% O_2_, at day 7 of adulthood, α-syn expression caused 38.1% of animals to have completely lost at least one DA neuron, compared to 8.75% in WT animals (Fig. 5a,b and Extended Data Fig. 4). One percent O_2_ reduced α-syn-induced neurodegeneration by half, demonstrating a protective effect (Fig. 5b). Interestingly, the level of α-syn-induced neurodegeneration at 1% O_2_ was not significantly different from WT animals grown in 1% O_2_, suggesting that 1% O_2_ may increase basal-age-related DA neurodegeneration. Taken together, the *C. elegans* results further support our model that α-syn expression induces MCI dysfunction that can be alleviated by hypoxia.

### Hypoxia reverses parkinsonism and halts loss of DA neurons

**Fig. 6:**
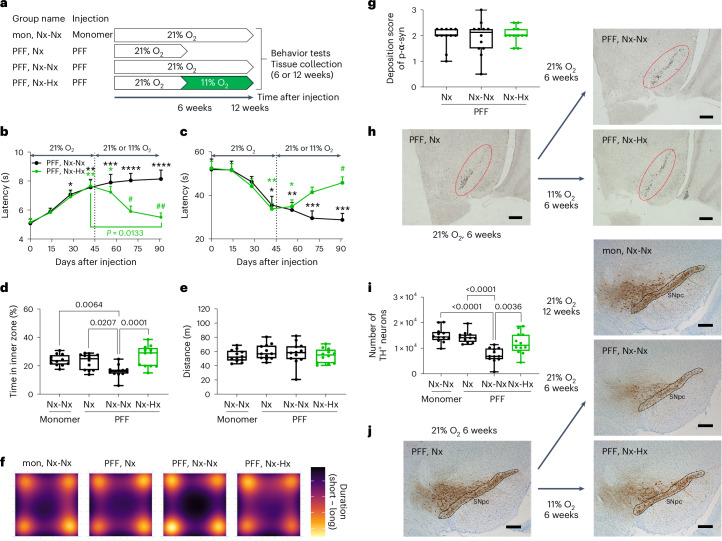
Breathing 11% O_2_ starting 6 weeks after PFF injection reverses movement disorders and further loss of DA neurons in the SNpc. **a**, Schematic depicting the experimental groups and time course. **b**,**c**, Results of pole (**b**) and cage hang (**c**) tests in mice that had a PFF injection in the ‘PFF, Nx-Nx’ and ‘PFF, Nx-Hx’ groups. *n* = 12 in each group. **b**,**c**, A two-way ANOVA followed by Tukey’s correction for post-hoc comparisons was conducted. Data are presented as the mean + s.e.m. *P < 0.05, **P < 0.01, ***P < 0.001 or ****P < 0.0001 versus day 0 in the same group, or ^#^P < 0.05 or ^###^P < 0.001 versus the ‘PFF, Nx-Nx’ group at the same time point. **d**,**e**, Time in the inner zone (**d**) and the total distance (**e**) in the OFT in mice that were injected with the α-syn monomer or PFF in the ‘mon, Nx-Nx’, ‘PFF, Nx’, ‘PFF, Nx-Nx’ and ‘PFF, Nx-Hx’ groups. *n* = 12 in each group. **d**,**e**, A one-way ANOVA followed by Tukey’s correction for post-hoc comparisons was conducted. **f**, Heatmap showing the location of mice during the OFT. Each image shows the average latency of mice in each group. **g**, Deposition score of phosphorylated α-syn in the SNpc in mice that were treated with PFF in the ‘PFF, Nx’, ‘PFF, Nx-Nx’ and ‘PFF, Nx-Hx’ groups. *n* = 12 in each group. **h**, Representative photomicrograph of the SNpc stained for phosphorylated α-syn in mice injected with PFF in the ‘PFF, Nx’, ‘PFF, Nx-Nx’ and ‘PFF, Nx-Hx’ groups. *n* = 12 in each group. **i**, Number of TH^+^ neurons in the SNpc in mice treated with α-syn monomer or PFF in the ‘mon, Nx-Nx’, ‘PFF, Nx’, ‘PFF, Nx-Nx’ and ‘PFF, Nx-Hx’ groups. *n* = 12 in each group. **j**, Representative photomicrograph of the SNpc stained for TH in mice injected with α-syn monomer or PFF in the ‘mon, Nx-Nx’, ‘PFF, Nx’, ‘PFF, Nx-Nx’ and ‘PFF, Nx-Hx’ groups. *n* = 12 in each group. Nx, normoxia (breathing 21% O_2_); Hx, hypoxia (breathing 11% O_2_). **g**,**i**, A one-way ANOVA followed by Tukey’s correction for post-hoc comparisons was conducted. **d**,**e**,**g**,**h**, Data are presented as box plots showing the median and IQR, with the whiskers denoting the maximum and minimum values. **h**,**j**, Scale bars, 100 μm. Source data

**Extended Data Fig. 5:**
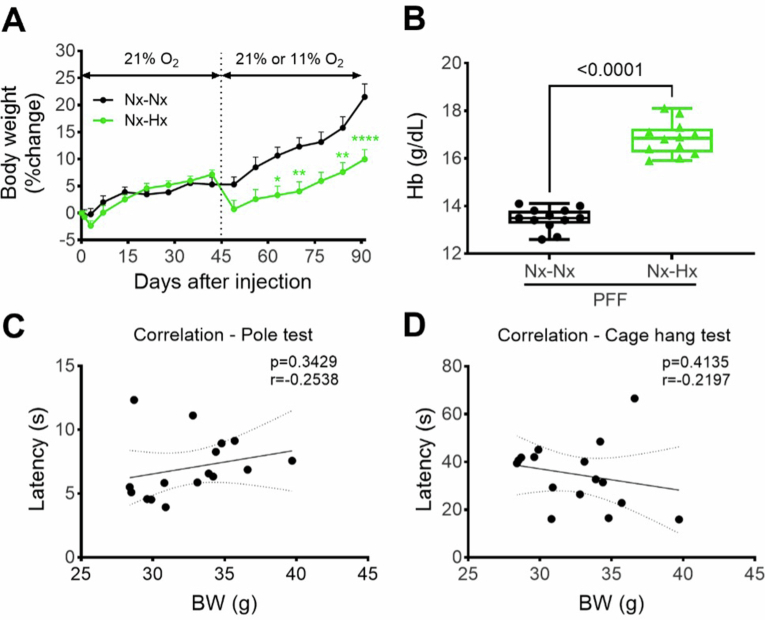
Impact of PFF injection and hypoxia treatment on body weight, hemoglobin concentration and performance of behavior tests. Changes in body weight (**a**) and hemoglobin concentration (**b**) in mice breathing 21% or 11% O_2_ for 12 weeks after injection of monomer or PFF. N = 12 in each group. Two-way ANOVA followed by Tukey’s correction for post-hoc comparisons was conducted for panel (**a**). Two-tailed t-test was conducted for panel (**b**). The Pearson correlation coefficient (r) with two-tailed hypothesis was calculated for panel (**c**) and (**d**). N = 16. Source data

Having observed the prevention of PFF-induced neurotoxicity and motor deficits by breathing 11% O_2_, we next investigated whether normobaric hypoxia could halt or even reverse the loss of DA neurons and the progression of movement disorders. For this experiment, the first group of mice received α-syn monomer in the right striatum and breathed 21% O_2_ for 12 weeks (monomer, normoxia-normoxia (mon, Nx-Nx)). Other mice received PFF injection in the right striatum and breathed 21% O_2_ for 6 weeks; then, they were divided into three groups (Fig. 6a). The second group was euthanized at 6 weeks after PFF injection for tissue collection (PFF, Nx). The third group continued to breathe 21% O_2_ for another 6 weeks for a total of 12 weeks (PFF, Nx-Nx). The fourth group breathed 11% O_2_ starting 6 weeks after PFF injection for another 6 weeks (PFF, normoxia-hypoxia (Nx-Hx)). The pole and cage hang tests were performed every 2 weeks in ‘PFF, Nx-Nx’ and ‘PFF, Nx-Hx’ mice for 12 weeks until euthanized for tissue collection. In addition, the OFT was conducted at 12 weeks after injection in ‘mon, Nx-Nx’, ‘PFF, Nx-Nx’ and ‘PFF, Nx-Hx’ mice, and at 6 weeks after injection in ‘PFF, Nx’ mice. ‘PFF, Nx-Nx’ and ‘PFF, Nx-Hx’ mice similarly developed movement disorders 6 weeks after PFF injection while breathing 21% O_2_ (Fig. 6b,c). However, while motor function continued to worsen in ‘PFF, Nx-Nx’ mice, it returned to baseline in ‘PFF, Nx-Hx’ mice during the subsequent 6 weeks of breathing 11% O_2_. In the OFT, the time spent in the inner zone did not change in mice that breathed 21% O_2_ for 6 weeks (PFF, Nx), but markedly decreased in mice that continued to breathe 21% O_2_ for 12 weeks after PFF injection (PFF, Nx-Nx). In contrast, breathing 11% O_2_ from 6 weeks after PFF injection prevented the reduction of the time spent in the inner zone (PFF, Nx-Hx) (Fig. 6d). The total distance covered by mice was not affected by PFF injection or breathing 11% O_2_ (Fig. 6e,f). Breathing 11% O_2_ starting 6 weeks after PFF injection decreased body weight and increased hemoglobin levels in ‘PFF, Nx-Hx’ mice compared to ‘PFF, Nx-Nx’ mice (Extended Data Fig. 5a,b). There was no correlation between body weight and the results of the pole and cage hang tests (Extended Data Fig. 5c,d). The number of LB-like inclusions in the SNpc increased similarly among the three groups that received PFF, suggesting that the number of LB-like inclusions had already plateaued 6 weeks after PFF injection (Fig. 6g,h). The number of TH^+^ neurons in the SNpc did not change in mice that breathed 21% O_2_ for 6 weeks, but significantly decreased in mice that continued to breathe 21% O_2_ for 12 weeks after PFF injection (PFF, Nx-Nx) compared to monomer-treated mice that breathed 21% O_2_ (mon, Nx-Nx) (Fig. 6i,j). In contrast, breathing 11% O_2_ starting 6 weeks after PFF injection (PFF, Nx-Hx) mitigated loss of TH^+^ neurons. These results suggest that breathing 11% O_2_ after the development of movement disorders and formation of LB-like inclusions can reverse motor symptoms and prevent further loss of TH^+^ dopamine neurons in the SNpc after PFF injection.

## Discussion

The current study reports that breathing chronic normobaric hypoxia (11% O_2_) not only prevents but also reverses motor deficits in a PD mouse model subjected to striatal injection of α-syn PFF. Immediate initiation of hypoxia treatment upon PFF injection prevents loss of TH^+^ DA neurons. Delayed initiation of hypoxia treatment halts neuronal loss, which is sufficient to restore motor function and prevent the development of anxiety-like behavior in PFF-injected mice. These observations suggest that it is possible to reverse motor and non-motor symptoms even if the treatment is started after LB-like inclusions have accumulated in the SNpc and symptoms become clinically evident, a situation that more closely recapitulates a treatment scenario in human PD. To the best of our knowledge, hypoxia is the only intervention that not only prevents, but also reverses, neurological defects in this model.

Hypoxia does not prevent the formation of α-syn aggregates. Rather, hypoxia confers neuroprotection in the face of these aggregates. This hypothesis is supported by the remarkable reversal of motor symptoms and prevention of further loss of DA neurons by breathing 11% O_2_ from 6 weeks after PFF injection, when the number of LB-like inclusions had already plateaued at the maximum level detected. Reducing α-syn levels or targeting α-syn aggregates has been suggested as a potential therapeutic target. However, there are concerns that reducing levels of α-syn can have detrimental consequences because of its physiological role in the synaptic vesicle endocytic and exocytic cycles. The data in this study suggest a treatment according to which loss of dopamine neurons can be prevented without having to target α-syn aggregation.

The ability of hypoxia to protect against the toxicity of α-syn is evolutionarily conserved in *C. elegans* overexpressing α-syn. While breathing 1% O_2_ on its own increased basal-age-related DA neurodegeneration, in the context of chronic hypoxia, overexpressed α-syn does not cause further toxicity on DA neurons. This result is analogous to what we have reported in the context of certain complex I mutants^22^ and frataxin loss^41^; both of these sets of mutants are also rescued by hypoxia in worms and in *C. elegans*. We have proposed that the ability of hypoxia to rescue certain mutants is analogous to the concept of temperature sensitivity^42^; by analogy, we find that in normoxia, α-syn aggregates form and are toxic to neurons, but that hypoxia creates a ‘permissive’ environment in which α-syn aggregates are no longer toxic, that is, α-syn aggregates and hypoxia are epistatic with each other.

While the precise mechanisms responsible for the toxicity of α-syn aggregates to neurons remain an unsolved problem, many prior studies have shown that PFF-induced α-syn aggregation compromises the activity of the mitochondrial respiratory chain in DA neurons of the SNpc^11,43^. Because more than 90% of oxygen is consumed by mitochondria, unused oxygen is theorized to accumulate when oxidative phosphorylation is impaired. Classic studies have shown that patients with inherited mitochondrial disease can exhibit tissue hyperoxia because of poor oxygen extraction, especially during exercise^32^. In fact, we previously reported hyperoxia in the vestibular nucleus and striatum of the *Ndufs4*^−/−^ mice breathing 21% O_2_. In this study, we uncovered that α-syn aggregation induced brain tissue hyperoxia in the SNpc of mice. It will be interesting to determine whether brain hyperoxia is also a feature of human PD. Although tissue hyperoxia could be a direct consequence of respiratory chain impairment and oxygen use, we acknowledge that there could be other mechanistic bases (for example, changes in vascularity, low neuronal and hence metabolic activity, changes in pH leading to more O_2_ offloading). Regardless, the brain tissue hyperoxia observed in the SNpc of mice breathing 21% O_2_ that also had PFF-induced α-syn aggregation supports the notion that high oxygen itself may be mediating the neurotoxic effects of α-syn aggregates.

α-Syn aggregation induces neuronal iron accumulation in the SNpc^35^. Our RNA-seq results, which revealed upregulation of *Tfrc* and *Fpn* by PFF and breathing 11% O_2_, support the potential role of iron accumulation in PD pathogenesis. Along this line, a recent study using a nonhuman primate model of PD reported that intranasal administration of α-syn PFF increased iron deposition and protein levels of TFR1 (encoded by *Tfrc*) and FPN in DA neurons in the SN of *Macaca fascicularis*^44^. We speculate that the combination of iron accumulation and tissue hyperoxia can jointly lead to neuronal toxicity via Fenton chemistry, which contributes to lipid peroxidation. IRP2 is a key posttranscriptional regulator in the iron starvation pathway including TFR1 and FPN. We previously reported that genetic ablation of FBXL5, the ubiquitin ligase that targets IRP2 for degradation, is tolerated in hypoxia but not normoxia^41^. Hence, activation of this pathway in normoxia is detrimental. Moreover, recent CRISPR genetic screens and cancer literature revealed that cells with compromised MCI are particularly susceptible to loss of GPX4, the key phospholipid hydroperoxidase that guards against death caused by lipid peroxidation^33,34^. The inhibition of tissue hyperoxia and lipid peroxidation by breathing 11% O_2_ for 6 weeks in PFF-treated mice further supports the critical pathogenic role of tissue hyperoxia that is probably induced by mitochondrial dysfunction.

In addition to directly attenuating brain tissue hyperoxia, the protective effects of hypoxia breathing may be mediated by inhibition of lipid peroxidation through the activation of HIF-dependent signaling. In fact, strong upregulation of HIF targets was observed in our RNA-seq and corroborated using either TMT proteomics or RT–qPCR in separate cohorts of mice that breathed hypoxia for 3 or 10 months. A recent study showed that HIF1α-induced lactate contributes to the resistance to lipid peroxidation of solid tumors in a pH-dependent manner^36^. Upregulation of *Ldha* and *Slc16a3* (*Mct4*) support this hypothesis given their roles in lactate generation and transport. We also note that the HIF targets *Ndufa4l2* is elevated by hypoxia, according to evidence both at the RNA and protein level. It has been reported to modulate mitochondrial electron transport chain activity and ROS production as a protective mechanism^37^. Our data support the notion that both HIF-dependent and HIF-independent mechanisms may be at play in conferring the observed neuroprotection.

This study has some limitations. We used the entire SN tissue, not the SNpc, for our RNA-seq, TMT proteomics, RT–qPCR and MDA assay because of the technical difficulty of selectively collecting the SNpc from mouse brains. Although DA neurons are concentrated in the SNpc, it is possible that the signals we observed may not reflect changes specific to DA neurons. Future studies using single-cell or single-nucleus transcriptomic analysis may enable further characterization of the mechanism of hypoxia rescue. In addition, unbiased transcriptomic analysis was performed after a relatively short follow-up (that is, 12 weeks) after PFF injection in the current study. A longer follow-up after PFF injection may reveal additional pathological signals, such as neuroinflammation and blockade of autophagy^45^. Lastly, efficacy and the possible untoward effects of hypoxia remain to be further examined especially in models of age-dependent neurodegeneration.

Our work may help to reconcile longstanding epidemiological observations for PD. One of the strongest (and troubling) epidemiological associations for PD is the protective effects of long-term, heavy smoking on the risk of developing PD^46^. Although multiple mechanisms have been put forward, we note that heavy smoking is associated with elevation in carbon monoxide (CO) levels (evidenced by high carboxyhemoglobin), which can slow down oxygen delivery. In fact, we have previously shown that sublethal doses of CO are protective in genetic MCI models of Leigh syndrome^47^. It is tempting to speculate that one of the reasons why heavy smoking is protective is because of mild reduction of oxygen delivery induced by smoking CO.

There is growing interest in the role of hypoxia in PD, both as a pathogenic mechanism and as a potential therapy^48^. The beneficial effects of hypoxia breathing in PD have been suggested based on a few anecdotal reports of patient experiences at high altitude^23^ and hypothetical benefits of intermittent hypoxia breathing in other neurological disorders, presumably via the activation of HIF signaling^49,50^. Along the line of the latter, a protocol for a clinical trial to study the effects of intermittent hypoxia breathing in individuals with PD has been published^51^. Because our RNA-seq and proteomics results showed upregulation of several HIF-dependent genes in the SN of mice breathing 11% O_2_, it is possible that the beneficial effects of hypoxia breathing are partially mediated via HIF-dependent mechanisms. Alternatively, it is also conceivable that chronic continuous hypoxia breathing prevents and reverses the progression of PD pathology through distinct mechanisms (for example, mitigating brain tissue hyperoxia) from intermittent hypoxia breathing. Our current study provides timely evidence showing robust neuroprotective effects of chronic continuous hypoxia breathing in a mouse model of PD induced by a templated conversion of endogenous α-syn to inclusions. Because chronic continuous hypoxia has been shown to be beneficial also in mouse models of Friedreich’s ataxia^41^, multiple sclerosis^52^ and premature aging^53^, it is likely to be broadly neuroprotective via multiple mechanisms. Further preclinical studies are needed to determine the safety and efficacy of chronic hypoxia breathing, including the optimal concentrations of hypoxic gas mixtures, longer durations of exposure and the effects of hypoxia at different disease stages. These results motivate more research into the effects of chronic continuous hypoxia breathing^42^, or pharmacological strategies that reduce oxygen delivery^54^, potentially leading to new therapeutic strategies to prevent and even halt the progression of PD.

## Methods

### Animals

All animal experimentation was approved by the Massachusetts General Hospital Institutional Animal Care and Use Committee. We used 14–17-week-old, age-matched and weight-matched male C57BL/6J mice (Jackson Laboratory). Mice had free access to food and water and were maintained in a 12-h light–dark cycle, at a temperature between 20 and 25 °C and humidity between 40% and 60%. The design of experiments involving animals followed the Animal Research: Reporting of In Vivo Experiments guidelines. To minimize variability, we used a randomized paired (matched pairs) design.

### Preparation of α-syn monomer and PFF

Mouse α-syn synthesis and purification was performed at the University of Alabama at Birmingham as described previously^55^. The α-syn protein underwent endotoxin removal with Pierce High-Capacity Endotoxin Removal Resin (Thermo Fisher Scientific). The amount of endotoxin in the purified α-syn was determined using the Pierce Chromogenic Endotoxin Quant Kit (Thermo Fisher Scientific) and confirmed to be below the threshold of 5 EU mg^−1^. The concentration of monomeric α-syn was measured using absorbance at 280 nm with an extinction coefficient of 7,450 M^−1^cm^−1^. Fibrils were generated by incubating monomeric α-syn (300 μM, 5 mg ml^−1^) in 150 mM KCl and 50 mM Tris-HCl at 37 °C with constant agitation for 7 days^56^. On the seventh day, fibrils were isolated from the starting material using centrifugation, removal of the supernatant and resuspending the pellet in approximately half the initial starting volume of buffer. To determine the resulting concentration of α-syn, 5 μl of fibrils were incubated with 8 M guanidinium chloride to dissociate the fibrils into monomer; the concentration was measured via NanoDrop. Fibrils were brought to 5 mg ml^−1^ with the aforementioned buffer and 25-μl aliquots of fibrils were snap-frozen and stored at −80 °C until use. Before injecting, the fibril aliquots were thawed and sonicated in volumes of 22–25 μl per tube using a Qsonica 700 W cup horn sonicator system. The pre-optimized sonication program runs at 30% amplitude for 15 min in a 3 s ON/2 s OFF cycle, for a total sonication time of 30 min. A recirculating water chiller system with filter was used to maintain the temperature of the water bath during sonication at 15 °C. The size of sonicated fibrils was measured to ensure proper fragmentation^28^ using dynamic light scattering on a DynaPro NanoStar Dynamic Light Scattering Detector (Wyatt Technology). Sufficient fragmentation of the fibrils is essential in the fibril model to induce α-syn seeding and subsequent formation of α-syn inclusions. Using a sample of the sonicated PFFs diluted 1:500 in 1× Dulbecco’s PBS, five measurements consisting of ten averaged acquisitions each were calculated to assess sample quality and estimate fibril fragment length. The desired size range of the fibrils for optimal seeding was between 20 and 70 nm. The average size of the sonicated fibrils for each experiment was between 25 and 50 nm. Sonicated fibrils were snap-frozen in 25-μl aliquots and shipped on dry ice overnight to Harvard where they were kept at −80 °C until the day of use. Before injection of monomeric α-syn, protein was thawed on ice, spun at 20,000*g* at 4 °C and kept on ice until injected. Only the supernatant from the α-syn monomer was injected and any post-centrifugation pellet was avoided.

### Injection of α-syn monomer and PFF

At 3–4 months of age, mice were deeply anesthetized with isoflurane on a stereotaxic frame (MM-8000/3, ASI instruments). Animals were unilaterally injected with 2 μl of 300 μM sonicated fibrils, or 300 μM monomeric α-syn as a control, into the right striatum. Solutions were injected at a constant rate of 0.5 μl min^−1^ and the needle was left in place for 2 min; this was followed by slowly withdrawing the needle. The coordinates for the right striatum were +0.2 mm anteroposterior, ±2.0 mm mediolateral and −3.2 mm dorsoventral. All mice survived after stereotaxic injection of α-syn monomer or PFF and were used for the following experiment with breathing 21% or 11% O_2_.

### Breathing 21% or 11% O_2_

Mice that breathed 21% or 11% O_2_ were housed in cages kept inside transparent acrylic boxes. An FiO_2_ of 11% was generated using an N_2_ generator (MAG-20, Higher Peak). O_2_ levels were monitored daily using an O_2_ sensor (MiniOX 1, Ohio Medical Corporation). CO_2_ concentrations inside the chamber were kept below 0.8% (monitored using an Extech CO200 Monitor, Extech Instruments) using soda lime (Sodasorb, Smiths Medical). Temperature (24–26 °C), humidity (30–70%) and light cycle (12 h light–dark) were maintained. Mice were allowed to freely access food and drinking water bottles. To examine whether hypoxia treatment prevented neurodegeneration and behavior disorders after injection of PFF, mice started to breathe 21% or 11% O_2_ in a chamber 1 h after the injection of α-syn monomer or PFF (Fig. 1a). To examine whether hypoxia treatment halted or reversed neurodegeneration and behavior disorders, mice were housed in a chamber with 21% O_2_ for 6 weeks starting 1 h after the injection of α-syn monomer or PFF and were either kept breathing 21% O_2_ or transferred to a chamber with 11% O_2_ for the following 6 weeks. Eleven cohorts of mice were studied as shown in Supplementary Table 1. Three mice in the 10-month cohort were euthanized because of limb injury from fighting after injection of PFF and were excluded from the statistical analysis.

### Immunohistochemistry

Mice were anesthetized with isoflurane and perfused with ice-cold 4% paraformaldehyde in PBS via the left ventricle to collect whole brains. After further fixing with 4% paraformaldehyde in PBS for 24 h, brains were sent to NeuroScience Associates. Brains were treated overnight with 20% glycerol and 2% dimethylsulfoxide to prevent freeze artifacts. Specimens were then embedded in a gelatin matrix using the MultiBrain/MultiCord technology (NeuroScience Associates). Blocks were rapidly frozen, after curing by immersion in 2-methylbutane chilled with crushed dry ice and mounted on a freezing stage of an AO 860 sliding microtome. The MultiBrain/MultiCord blocks were sectioned coronally with a 40-μm setting on the microtome. All sections were cut through the entire length of the specimen segment and collected sequentially into series of 24 containers. All containers contained Antigen Preserve solution (50% PBS, pH 7.0, 50% ethylene glycol, 1% polyvinylpyrrolidone); no sections were discarded. For immunohistochemistry, free-floating sections were stained with the desired stain. All incubation solutions from the primary antibody onward used Tris-buffered saline with Triton X-100 as the vehicle; all rinses were with Tris-buffered saline.

After hydrogen peroxide treatment, sections were immunostained with the primary antibodies for α-syn phosphorylated at Ser129 (1:18,000 dilution, cat. no. 010-26481, Wako Chemicals) or TH (1:15,000 dilution, cat. no. p40101, Pel-Freez Biologicals) overnight at room temperature. Vehicle solutions contained Triton X-100 for permeabilization. After the rinses, a biotinylated secondary antibody (1:1,000 dilution for TH, cat. no. BA-1000, Vector laboratories) was applied. We did not use the secondary antibody for α-syn phosphorylated at Ser129 because the primary antibody was already biotinylated. After further rinses, Vector Laboratories ABC solution (avidin–biotin–horseradish peroxidase complex; details in the instructions for VECTASTAIN Elite ABC, Vector Laboratories) was applied. Sections were again rinsed, then treated with DAB with hydrogen peroxide for TH or DAB with hydrogen peroxide and nickel(II) sulfate for α-syn phosphorylated at Ser129 to create a visible reaction product. After further rinses, sections were mounted on gelatin-coated glass slides, air-dried and run through the following sequences for Nissl counterstaining. For the thionine Nissl counterstain on TH, slides were fixed with 95% ethanol/formaldehyde and then rehydrated before incubation in thionine (thionine/acetate buffer, pH 4.5). For Neutral Red counterstaining on α-syn pSer129, slides were rehydrated and then placed into the Neutral Red solution (neutral red/acetate buffer, pH 4.5). After counterstaining, slides were dehydrated in alcohol, cleared in xylene and coverslipped using Permount Mounting Medium. Bright-field images were acquired using an Eclipse 80i microscope (Nikon Instruments). Image analysis was performed by an experimenter blinded to the identity of the images analyzed using ImageJ v.1.53k. The deposition score of phosphorylated α-syn in SN was evaluated using previously reported scoring criteria^28^. Results are reported as the average of the analyzed 2–4 sections of each biological replicate. To assess the loss of DA neurons in the SNpc, the number of TH^+^ and Nissl^+^ cells in the SN was counted using stereological analysis as described below.

### Stereological analysis

Unbiased stereology was conducted to count TH^+^ and Nissl^+^ neurons in the SNpc region by a researcher blinded to the experimental conditions and groups. Quantitative analysis was performed using an Olympus BX51 bright-field microscope to examine 40-μm-thick brain sections containing the SNpc. To identify the SNpc, we used the Paxinos Mouse Brain Atlas^57^. Sections at the anteroposterior coordinates, relative to bregma, were between −2.92 mm and −3.64 mm. An average of six sections containing the area of interest, spaced 160 μm apart, were included and stained to identify TH^+^ cells in the SNpc. Quantification was performed using an Optical Fractionator probe (Stereo Investigator v.2021, MBF Bioscience). The sampling parameters for each section were established as follows: a section thickness of 40 µm and an evaluation interval of six sections. At a magnification of ×4, the contours of the SNpc were traced using the freehand tool, with the stained TH DA cells serving as references. At ×40 magnification, the remaining parameters necessary for stereological analysis were set: a counting frame of 50 × 50 µm, a mounted thickness of 33 µm, a grid size of 100 × 100 µm and an optical dissector height of 22 µm. Quantification variability, as assessed using the Schmitz–Hof coefficient of error, was below 0.270 for all animals across experimental groups, with a median of 0.065 and a mean of 0.0737.

### Behavioral evaluation

Behavioral tests were conducted between 8:00 and 12:00 for the pole test and the cage hang test or 12:00 and 17:00 for the OFT considering circadian rhythm, as reported previously^29,58^. To conduct the pole test, a vertical metal pole with 2-cm diameter and 40-cm length was placed on the floor of a housing cage. Mice were placed on the top of the vertical metal pole and the time needed to reach the floor was measured. To conduct the cage hang test, a metal wire frame of the housing cage (WBL7115SMD-AMG, Allentown) was placed approximately 40 cm above the cage floor. Mice were hung on the metal wire frame and the time needed to drop onto the cage floor was measured. Each test was conducted five times and the average of the last three trials was used for the analysis. We performed the OFT to evaluate motor function and behavior across treatment groups. The OFT apparatus consisted of four boxes measuring 50 × 50 cm each. In addition to the opaque black walls of each field, the whole apparatus was surrounded by a 1.5-m wall to reduce outside interferences, such as air drafts or visual distractions. An indirect light source was used to ensure even and dim illumination of all fields. Mice were kept in the procedural room for at least 60 min before testing. At the start of the test, one mouse per box was put into the center of the open field. A maximum of four mice were tested simultaneously. Mice were then allowed to explore the field for 15 min. During the test, an overhead camera was used to record each mouse with the ANY-maze software v.7.3 (Stoelting). The ANY-maze software was used to automatically track and analyze the animals’ positions. All experimenters left the room for the duration of the test after starting the recording. Before and after each test run, all surfaces of the apparatus were wiped with 70% ethanol and left to air-dry. Experimenters were blinded to the treatment groups. Heatmaps showing integration of the movement trace from all mice in each group were created with R v.4.3.2 (packages: tidyverse, ggpubr).

### Detection of iron

To detect ferric and ferrous iron in the brain, Perls-DAB staining was performed^59^. Three sections containing the SNpc were processed for each biological replicate. Sections were rinsed in deionized H_2_O (dH_2_O) then placed in a freshly prepared (1:1) 2% HCl and 2% potassium ferrocyanide solution. Sections were again rinsed, then treated with a DAB solution (0.05% DAB and 0.011% hydrogen peroxide) to amplify the Perls reaction product. After further rinses, sections were mounted on gelatin-coated glass slides, then air-dried. The mounted slides were then counterstained with a light thionine (Nissl) counterstain. For the Nissl counterstain, air-dried mounted stained slides were carried through the following sequence: 95% ethanol; 95% ethanol/formaldehyde; 95% ethanol; 70% ethanol; dH_2_O. Slides were then stained in a thionine solution made in an acetate buffer (pH 4.5), rinsed in dH_2_O and visually assessed. After the dH_2_O rinses, slides were dehydrated in alcohol, cleared in xylene and coverslipped with Permount Mounting Medium. Bright-field images were acquired using an Eclipse 80i microscope. Image analysis was performed by an experimenter blinded to the identity of the images analyzed using the QuPath (v.0.3.2) software. Color deconvolution was performed to separate Nissl from the Perls-DAB signal^60^. The SNpc area was manually annotated by an experimenter blinded to the identity of the images using a brain atlas of the mouse as a ref. ^57^. Perls-DAB was quantified by thresholding the DAB signal using ImageJ (v.1.53k), with a threshold of 220. The Perls-DAB signal was normalized to the SNpc area. Results are reported as the average of the analyzed three sections of each biological replicate.

### Brain tissue *p*O_2_ in the SNpc

Mice were anesthetized with isoflurane (induction at 2–4%, maintenance at 1–1.5%) in 21% O_2_. Rectal temperature was kept at 37 °C using a heating pad seated under the mouse during the whole procedure. Mice were placed in a prone position with the head stabilized using a stereotaxic frame. After incision and dissection of the skin, an opening in the skull was created on the ipsilateral side of the PFF or monomer injection using a Micro-Drill (MD-1200, Braintree Scientific). Then, the optical *p*O_2_ probe (OxyLab, Oxford Optronix) was inserted to detect the brain tissue *p*O_2_ in the SNpc. The coordinates were mediolateral = 1.20 mm, anteroposterior = −3.20 mm and dorsoventral = −4.40 mm from bregma. During the brain *p*O_2_ measurement, the depth of anesthesia was reduced by lowering the isoflurane concentration to 0.75% to minimize the impact of anesthesia on brain *p*O_2_.

### Detection of MDA

We measured the concentration of MDA in the SN to evaluate the effect of hypoxia on lipid peroxidation using a commercially available kit (cat. no. ab118970, Abcam). The assay was conducted according to the manufacturer’s protocol.

### RNA-seq

RNA extraction, library preparation and sequencing was conducted at Azenta Life Sciences. Total RNA was extracted from dissected fresh-frozen SN samples using the QIAGEN RNeasy Plus Universal Mini Kit according to the manufacturer’s instructions. RNA samples were quantified using the Qubit 2.0 Fluorometer (Thermo Fisher Scientific) and RNA integrity was checked using the Agilent Technologies TapeStation 4200. RNA sequencing libraries were prepared using the NEBNext Ultra RNA Library Prep Kit for Illumina using the manufacturer’s instructions (New England Biolabs). Briefly, mRNAs were initially enriched with Oligod(dT) beads. Enriched mRNAs were fragmented for 15 min at 94 °C. First-strand and second-strand complementary DNA (cDNA) was subsequently synthesized. cDNA fragments were end-repaired and adenylated at the 3′ ends; universal adapters were ligated to the cDNA fragments, followed by index addition and library enrichment using PCR with 13 cycles. The sequencing library was validated on the Agilent TapeStation and quantified using the Qubit 2.0 Fluorometer, as well as by qPCR (KAPA Biosystems). The sequencing libraries were clustered on a flowcell. After clustering, the flowcell was loaded on the Illumina instrument (4000 or equivalent) according to the manufacturer’s instructions. Samples were sequenced using a 2× 150-bp paired-end configuration to at least 20 million reads per sample (median = 27 M reads). Image analysis and base calling were conducted using the Control software. Raw sequence data (.bcl files) generated by the sequencer were converted into .fastq files and demultiplexed using the Illumina bcl2fastq v.2.17 software. One mismatch was allowed for index sequence identification.

### RNA-seq data analysis

Read quality was verified using FastQC^61^ (v.0.11.9); then, the reads were processed using a custom bulk RNA-seq processing pipeline. First, any rDNA-derived transcripts remaining after poly(A) selection were filtered out by aligning the reads to the mouse rDNA reference and annotation (GenBank ID: BK000964.3) with STAR aligner^62^ (v.2.7.5a), keeping only the unaligned reads. After this in silico rRNA depletion step, STAR was used again to align the remaining reads to the mouse reference genome (mm10 with GENCODE annotation v.M24) with the parameters --outSAMattributes NH HI NM MD AS XS nM and --twopassMode Basic. Next, for any reads that mapped equally well to the nuclear and mitochondrial genome, mitochondrial alignment was set as the primary alignment. Then, duplicate reads were removed with the Picard MarkDuplicates tool^63,64^ (Picard v.2.21.9, as distributed in the Genome Analysis Toolkit v.4.1.5.0-9-g227bef6-SNAPSHOT); reads were assigned to genes using featureCounts (v.2.0.1)^65^ with the parameters -p, -M, and --primary. The SAMTools suite (v.1.12)^66^ was used throughout to manipulate, sort and filter the sequencing data in BAM format. After all samples made it through the pipeline, the read counts per gene were aggregated from the featureCounts output files into a single read count matrix with a row for each sample and a column for each gene for downstream analysis.

The raw count matrix was joined with the metadata about the samples and genes using anndata (v.0.7.5)^67^; scanpy (v.1.8.1)^68^ was used to conduct a principal components analysis (PCA). Raw counts were first normalized using TPM, then log-scaled with the scanpy.pp.log1p function. The gene list was filtered to only contain protein-coding genes; then, highly variable genes were identified with the scanpy.pp.highly_variable_genes function with min_mean = 1.25, max_mean = 7 and min_disp = 1.5. Finally, the normalized and logged counts were *z*-score-scaled using the scanpy.pp.scale function with max_value = 10; the scanpy.tl.pca function with svd_solver = ‘arpack’ was used to perform the PCA, using the scaled, highly variable genes as the features. This initial PCA highlighted some outliers, so an iterative procedure of removing outliers and updating the PCA was used until there were no outliers dominating the variance in principal components 1 or 2. Six of 48 samples were removed in this way.

**Extended Data Fig. 6:**
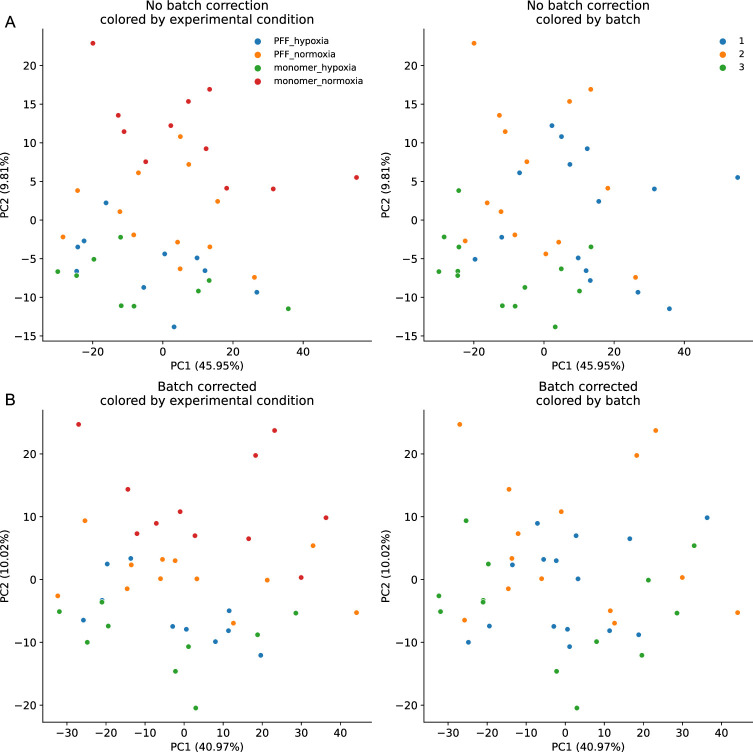
Mild RNA-seq batch effect is successfully mitigated after correction. Pairs of principal components analysis plots of the uncorrected data (**a**) and corrected data (**b**), with the left plot in each pair colored by experimental condition and the right plot in each pair colored by batch. Source data

In addition, the PCA analysis revealed a batch effect that was associated with the date on which mice were euthanized. The ComBat-seq^69^ tool (loaded with R v.4.0.5, via the sva v.3.38.0 package) was applied on the 42 non-outlier samples to adjust for the batch effect, while specifying the O_2_ status (hypoxia or normoxia) and injection type (PFF or monomer) as additional covariates to preserve the signal associated with our experimental variables. A final PCA on the batch-corrected count matrix returned by ComBat-seq confirmed that the batch effects were successfully mitigated (Extended Data Fig. 6).

After PCA, DESeq2 (v.1.30.1)^70^ was used to test all protein-coding genes for differential expression across our experimental conditions. This was accomplished by using the batch-corrected count matrix as the input and applying DESeq2 with a single categorical ‘experimental condition’ covariate that combined each sample’s oxygen status and injection type. The resulting DESeq2 model was used to compare the samples from each of our four experimental conditions: monomer air; monomer hypoxia; PFF air; and PFF hypoxia. Genes were called differentially expressed if they had a *P*_adj_ < 0.05 and log_2_ fold change > 0.25 (Supplementary Table 2).

### Mass spectrometry proteomics

Mass spectrometry proteomics experiments were performed by the ThermoFisher Center for Multiplexed Proteomics at Harvard Medical School, using methods that have been previously described for sample preparation, phosphopeptide enrichment, reverse phase separation, tandem mass spectrometry and protein identification^71–77^. More details on TMT sample acquisition and initial mass spectrometry data processing are available in the metadata of our PRIDE repository submission with the PXD063553 dataset identifier (see Data availability).

### TMT proteomics differential expression analysis

Column-sum-normalized data were assembled with the sample metadata into an annotated data object (AnnData v.0.9.1) in Python (v.3.10.11); Scanpy (v.1.9.3) was used for the initial analysis. Normalized data were log-transformed using scanpy.pp.log1p with default parameters; highly variable proteins were detected using scanpy.pp.highly_variable_genes, with min_mean = 0, max_mean = 10 and min_disp = 0.5; then, data were *z*-score-scaled using scanpy.pp.scale, with zero_center = True. Next, scaled data were used in the PCA, using scanpy.tl.pca, with n_comps = 15, random_state = 2 and use_highly_variable = True. Three samples, from three different experimental conditions, were identified as outliers in the first three principal components. We removed these three outliers, recomputed the PCA and verified that none of the other samples were outliers and that they clustered according to experimental condition when plotting principal component 1 versus principal component 2.

Next, we performed differential expression analysis using limma (v.3.54.0)^78^ in R (v.4.2.0). We loaded the column sum-normalized data into R, log_2_-transformed it after adding one to all values and applied a median normalization across samples using the limma’s function normalizeMedianValues. We used the lm.fit function to fit a linear model based on a simple formula of ~condition − 1, where condition is a factor containing four levels representing our experimental conditions: monomer treatment in normoxia; monomer treatment in hypoxia; PFF treatment in normoxia; and PFF treatment in hypoxia. We used limma contrasts to extract the differential expression results for individual comparisons, using the makeContrasts, contrasts.fit and eBayes functions for each comparison to create the contrast matrix, fit the model using that contrast and perform empirical Bayes smoothing, respectively (Supplementary Table 3).

### RT–qPCR

Total RNA was extracted from the SN tissues using TRIzol and phase separation, followed by column purification using the *Quick*-RNA Miniprep Kit (cat. no. R1055, Zymo Research)^79^. Reverse transcription was performed using the High-Capacity cDNA Reverse Transcription Kit (cat. no. 4374967, Invitrogen) according to the manufacturer’s instructions. qPCR reactions were performed using TaqMan and standard amplification protocols on the QuantStudio 5 Real-Time PCR System (Applied Biosystems). Gene expression was normalized to 18S or *Gapdh* and calculated using the ΔΔ^Ct^ method. TaqMan probes for 18S (Mm03928990_g1), *Gapdh* (Mm99999915_g1)*, Vegfa* (Mm01281449_m1), *Kdr* (Mm01222421_m1), *Adm* (Mm00437438_g1), *Iba1* (Mm00479862_g1), *Tfrc* (Mm01344477_g1), *Slc16a3* (Mm00446102_m1) and *Slc40a1* (Mm01254822_m1) were used for the qPCR reactions.

### *C. elegans*

Strains BY200: *Is[dat-1p::gfp]* and UA44: *baIn11[Pdat-1::alpha-syn* *+* *Pdat-1::gfp]* were maintained on nematode growth medium seeded with *Escherichia coli* strain OP50. Strains were provided by the *Caenorhabditis* Genetics Center, which is funded by the National Institutes of Health (NIH) Office of Research Infrastructure Programs (no. P40 OD010440). Animals were grown in 21% or 1% oxygen using a hypoxic in vitro cabinet (Coy Laboratory Products) at room temperature for many generations before the experiment. To measure DA neurodegeneration, 50 L4 stage animals were picked to fresh nematode growth medium plates; worms were moved to fresh plates every day until no longer producing progeny. Seven days after picking L4 stage animals, animals were picked into a 1-μl drop of sodium azide on a microscope slide with an agar pad (2% low electroendosmosis agarose in M9). Immediately after sodium azide paralysis, worms were gently covered with a coverslip and imaged and scored using a ZEISS Axio Imager Z1 microscope equipped with a ZEISS AxioCam HRc digital camera. GFP-tagged CEP neurons in the head of each animal were scored as ‘intact’, ‘degenerating’ or ‘missing’ based on the presence or absence of a clear cell boundary and intensity of GFP. Neurons were scored as ‘missing’ when not visible according to GFP. Representative images of intact and degenerating neurons in the 21% oxygen condition were captured. Approximately 30 worms were analyzed per condition and repeated in triplicate for a total of at least 80 worms scored per strain and per condition.

### Statistics and reproducibility

A one-way or two-way ANOVA followed by Tukey’s, Šídák’s or Dunnett’s correction for post-hoc comparisons was used. A Fisher’s exact test was conducted to compare two groups with a two-sided *P* value in the study of *C. elegans*. A two-tailed *t*-test was used to compare two groups. Compared groups are shown as box plots or mean values with s.e. A Pearson correlation coefficient (*r*) with two-tailed hypothesis was conducted for correlation. Significance was considered at *P* < 0.05. Prism v.10.3.1 (GraphPad Software) was used for the statistical analysis. Differential expression testing was conducted using DESeq2 (v.1.30.1 with R v.4.0.5) for the RNA-seq data, and limma (v.3.54.0 with R v.4.2.0) for the TMT proteomics data. To minimize variability, we used a randomized paired (matched pairs) design. We paired mice to two or more treatment groups on the basis of similar weight, age, delivery date and, when possible, holding cage. After pairing, mice were randomly assigned to the experimental groups by an investigator who did not perform the experiments. Investigators were blinded to group assignment when performing the behavioral tests, sample collections, DA neuron counting and histological analysis. No statistical methods were used to predetermine sample sizes but our sample sizes are similar to those reported in previous publications^28,80^. Data distribution was assumed to be normal but this was not formally tested. All individual data points are available in the Source data file. For the histological analysis, the mean value from multiple brain sections (2–4 sections for α-syn phosphorylated at Ser129 and Perls-DAB staining) in each biological replicate was used for the statistical analysis. For the stereological analysis to count TH^+^ neurons, 5–7 brain sections were analyzed in each biological replicate.

### Reporting summary

Further information on research design is available in the Nature Portfolio Reporting Summary linked to this article.

## Online content

Any methods, additional references, Nature Portfolio reporting summaries, source data, extended data, supplementary information, acknowledgements, peer review information; details of author contributions and competing interests; and statements of data and code availability are available at 10.1038/s41593-025-02010-4.

## Supplementary information

Supplementary Information Supplementary Tables 1–3.

Reporting Summary

Supplementary Tables 1–3. Supplementary Tables 1–3. Supplementary Table 1: Mouse cohorts. Supplementary Table 2: RNA-seq. Supplementary Table 3: TMT proteomics data

## Source data

Source Data Fig. 1 Statistical source data.

Source Data Fig. 2 Statistical source data.

Source Data Fig. 3 Statistical source data.

Source Data Fig. 4 Statistical source data.

Source Data Fig. 5 Statistical source data.

Source Data Fig. 6 Statistical source data.

Source Data Extended Data Fig. 1 Statistical source data.

Source Data Extended Data Fig. 2 Statistical source data.

Source Data Extended Data Fig. 3 Statistical source data.

Source Data Extended Data Fig. 5 Statistical source data.

Source Data Extended Data Fig. 6 Statistical source data.

## Data Availability

The RNA-seq data discussed in this publication have been deposited in the Gene Expression Omnibus^81^ and are accessible through accession no. GSE296779. The mass spectrometry proteomics data have been deposited in the ProteomeXchange Consortium via the PRIDE^82^ partner repository with the PXD063553 dataset identifier. The RNA-seq data were aligned to the mm10 mouse genome and transcript counts were assessed using the GENCODE v.M24 genome annotation. The mass spectra generated in the TMT proteomics experiment were searched against the mouse UniProt database downloaded in May 2021 (release 2021_04). The data supporting the findings of this study are available from the corresponding author upon reasonable request. Source data are provided with this paper.

## Code availability

The code used for the analysis and visualization of the bulk RNA-seq and TMT proteomics data is available in GitHub (https://github.com/MoothaLab/2025_marutani_pd_mouse). The code used to align the raw RNA-seq reads was published previously^53^ and is also available in GitHub (https://github.com/MoothaLab/ercc1-mouse-rob).
