# Supplementary material for: Hypoxia ameliorates neurodegeneration and movement disorder in a mouse model of Parkinson’s disease
Source: Nat Neurosci. 2025 Aug 6;28(9):1858–67. doi: 10.1038/s41593-025-02010-4 (PMC12411263; doi:10.1038/s41593-025-02010-4)
Supplement: Supplementary file 1 — Supplementary Tables 1–3. [file 41593_2025_2010_MOESM1_ESM.pdf]

nature neuroscience

Supplementary information https://doi.org/10.1038/s41593-025-02010-4

Hypoxia ameliorates neurodegeneration and movement disorder in a mouse model of Parkinson's disease

In the format provided by the authors and unedited

Table S1. Mouse cohorts (separate file).

Table S2. (separate file)

This table contains several sheets:
Read Me: Detailed explanation of entries in each data sheet
Sample metadata: This sheet contains metadata about the samples corresponding to the columns of the counts matrices.
Gene metadata: This sheet contains metadata about the genes corresponding to the rows of the counts matrices.
Raw counts: This sheet contains the raw count data for all samples, as produced by our RNA-seq processing pipeline.
Batch corrected raw counts: This sheet contains the raw counts after batch correction with the ComBat-seq tool. Note that this table omits samples that were outliers in an initial PCA based on the non-batch-corrected data.
Batch corrected TPM counts: This sheet contains the TPM-normalized count values based on the raw counts from the "batch corrected raw counts" sheet.
PFF norm vs monomer norm: The results of running DESeq2 on the "batch corrected raw counts" and then testing for differential expression between the PFF normoxia and monomer normoxia samples.
Monomer hyp vs monomer norm: The results of running DESeq2 on the "batch corrected raw counts" and then testing for differential expression between the monomer hypoxia and monomer normoxia samples.
PFF hyp vs monomer hyp: The results of running DESeq2 on the "batch corrected raw counts" and then testing for differential expression between the PFF hypoxia and monomer hypoxia samples.

Table S3. (separate file)

This table contains several sheets:
Read Me: Detailed explanation of entries in each data sheet
sample metadata: This sheet contains metadata about the samples corresponding to the columns of the counts matrices.
protein metadata: This sheet contains metadata about the genes corresponding to the rows of the counts matrices.
column sum norm all: This sheet contains the column sum-normalized protein intensities for all samples, as produced by the Thermo Fisher Center for Multiplexed Proteomics (TCMP) processing pipeline.
column sum norm outlier filt: This sheet contains the column sum norm data, but with samples that were outliers on principal components analysis (PCA) removed.
log2 median norm outlier filt: This sheet contains the column sum norm outlier filt data, but with additional log2-transformation and median-normalization. The median normalization was accomplished by the normalizeMedianValues function in limma.
PFF norm vs monomer norm: The results of running limma on the "log2 median norm outlier filt" data and then testing for differential expression between the PFF normoxia and monomer normoxia samples.

monomer hyp vs monomer norm: The results of running limma on the "log2 median norm outlier filt" data and then testing for differential expression between the monomer hypoxia and monomer normoxia samples.
PFF hyp vs monomer hyp: The results of running limma on the "log2 median norm outlier filt" data and then testing for differential expression between the PFF hypoxia and monomer hypoxia samples.
