# Supplementary material for: Hypoxia ameliorates neurodegeneration and movement disorder in a mouse model of Parkinson’s disease
Source: Nat Neurosci. 2025 Aug 6;28(9):1858–67. doi: 10.1038/s41593-025-02010-4 (PMC12411263; doi:10.1038/s41593-025-02010-4)
Supplement: Supplementary file 2 — Reporting Summary [file 41593_2025_2010_MOESM2_ESM.pdf]

nature portfolio

Corresponding author(s): Fumito ichinose
Last updated by author(s): 05/05/2025

Reporting Summary

Nature Portfolio wishes to improve the reproducibility of the work that we publish. This form provides structure for consistency and transparency in reporting. For further information on Nature Portfolio policies, see our Editorial Policies and the Editorial Policy Checklist.

Statistics

For all statistical analyses, confirm that the following items are present in the figure legend, table legend, main text, or Methods section.

n/a	Confirmed	
☐	☒	The exact sample size (*n*) for each experimental group/condition, given as a discrete number and unit of measurement
☐	☒	A statement on whether measurements were taken from distinct samples or whether the same sample was measured repeatedly
☐	☒	The statistical test(s) used AND whether they are one- or two-sided<br>*Only common tests should be described solely by name; describe more complex techniques in the Methods section.*
☒	☐	A description of all covariates tested
☐	☒	A description of any assumptions or corrections, such as tests of normality and adjustment for multiple comparisons
☐	☒	A full description of the statistical parameters including central tendency (e.g. means) or other basic estimates (e.g. regression coefficient) AND variation (e.g. standard deviation) or associated estimates of uncertainty (e.g. confidence intervals)
☐	☒	For null hypothesis testing, the test statistic (e.g. *F*, *t*, *r*) with confidence intervals, effect sizes, degrees of freedom and *P* value noted<br>*Give P values as exact values whenever suitable.*
☒	☐	For Bayesian analysis, information on the choice of priors and Markov chain Monte Carlo settings
☒	☐	For hierarchical and complex designs, identification of the appropriate level for tests and full reporting of outcomes
☐	☒	Estimates of effect sizes (e.g. Cohen's *d*, Pearson's *r*), indicating how they were calculated

Our web collection on statistics for biologists contains articles on many of the points above.

Software and code

Policy information about availability of computer code

Data collection

Provide a description of all commercial, open source and custom code used to collect the data in this study, specifying the version used OR state that no software was used.

Data analysis

Details of analysis is described in the Method section of the manuscript.

Statistical analysis for RNA-seq was conducted using Python v3.7.12, R v4.0.5, FASTQC v0.11.9, STAR aligner v2.7.5a, Picard tools v2.21.9, featureCounts v2.0.1, SAMTools v1.12, AnnData v0.7.5, Scanpy v1.8.1, ComBat-seq (implemented in R package sva v3.38.0), DESeq2 v1.30.1.

TMT proteomics initial data processing and protein identification was conducted using Monocle v0.4.43, Comet v2020.01 rev. 4, and AScorePro v1. Statistical analysis for TMT proteomics was conducted using Python v3.10.11, R v4.2.0, AnnData v0.9.1, Scanpy v1.9.3, and limma v3.54.0.

Statistical analysis for other experiment was performed using GraphPad Prism 10.3.1 (GraphPad Software, La Jolla, CA).

For manuscripts utilizing custom algorithms or software that are central to the research but not yet described in published literature, software must be made available to editors and reviewers. We strongly encourage code deposition in a community repository (e.g. GitHub). See the Nature Portfolio guidelines for submitting code & software for further information.

Data

Policy information about availability of data

All manuscripts must include a data availability statement. This statement should provide the following information, where applicable:

- Accession codes, unique identifiers, or web links for publicly available datasets
- A description of any restrictions on data availability
- For clinical datasets or third party data, please ensure that the statement adheres to our policy

The RNA-seq data discussed in this publication have been deposited in NCBI's Gene Expression Omnibus81 and are accessible through GEO Series accession number GSE296779 (https://www.ncbi.nlm.nih.gov/geo/query/acc.cgi?acc=GSE296779). The mass spectrometry proteomics data have been deposited to the ProteomeXchange Consortium via the PRIDE82 partner repository with the dataset identifier PXD063553 and doi 10.6019/PXD063553. RNA-seq data were aligned to the mm10 mouse genome reference, and transcript counts were assessed using the GENCODE vM24 genome annotation. The mass spectra generated in the TMT proteomics experiment were searched against the mouse Uniprot database downloaded in May 2021 (release 2021_04).

Research involving human participants, their data, or biological material

Policy information about studies with human participants or human data. See also policy information about sex, gender (identity/presentation), and sexual orientation and race, ethnicity and racism.

Reporting on sex and gender	The study did not involve human research participants.
Reporting on race, ethnicity, or other socially relevant groupings	The study did not involve human research participants.
Population characteristics	The study did not involve human research participants.
Recruitment	The study did not involve human research participants.
Ethics oversight	The study did not involve human research participants.

Note that full information on the approval of the study protocol must also be provided in the manuscript.

Field-specific reporting

Please select the one below that is the best fit for your research. If you are not sure, read the appropriate sections before making your selection.

☒ Life sciences ☐ Behavioural & social sciences ☐ Ecological, evolutionary & environmental sciences

For a reference copy of the document with all sections, see nature.com/documents/nr-reporting-summary-flat.pdf

Life sciences study design

All studies must disclose on these points even when the disclosure is negative.

Sample size	No statistical methods were used to pre-determine sample sizes but our sample sizes are similar to those reported in previous publications (doi:10.1074/jbc.RA119.007743) (doi.org/10.1016/j.nbd.2023.106287). A minimum sample size of 4 was used per group for experiments, as is standard and comparable to prior published experiments using similar techniques. The sample size is sufficient to detect a difference between two groups with coefficient of variation of 0.5 (ratio of standard deviation to mean difference) and two tailed level of significance (alpha) of 0.05 with a power of 80%.
Data exclusions	For RNA-seq, 6 samples out of 48 bulk RNA-seq samples were excluded as outliers in the initial PCA. For TMT proteomics, 3 samples out of 16 were excluded as outliers on PCA.
Replication	All experiments were performed at least twice and results were reliably reproduced. Independent biologic replicates are shown in all figures.
Randomization	We used randomized paired (a.k.a. matched pairs) design. We paired animals to two or more treatment groups on the basis of similar weight, age, delivery date, and when possible holding cage.
Blinding	In all assessment, investigators who determined the outcome was blinded to the group.

Reporting for specific materials, systems and methods

We require information from authors about some types of materials, experimental systems and methods used in many studies. Here, indicate whether each material, system or method listed is relevant to your study. If you are not sure if a list item applies to your research, read the appropriate section before selecting a response.

Materials & experimental systems

n/a	Involved in the study
☐	☒ Antibodies
☒	☐ Eukaryotic cell lines
☒	☐ Palaeontology and archaeology
☐	☒ Animals and other organisms
☒	☐ Clinical data
☒	☐ Dual use research of concern
☒	☐ Plants

Methods

n/a	Involved in the study
☒	☐ ChIP-seq
☒	☐ Flow cytometry
☒	☐ MRI-based neuroimaging

Antibodies

Antibodies used	Primary Antibody: Tyrosine Hydroxylase (Catalog#: p40101, Lot#: ajo3190, Pel Freez), Secondary Antibody for Tyrosine Hydroxylase: Anti-Rabbit Biotinylated (ba-1000, Vector)<br><br>Primary Antibody: Alpha Synuclein Phospho Ser129 (Catalog#: 010-26481, Lot#: CAK2272, FUJIFILM Wako Chemicals)<br>We did not use the second antibody for Alpha Synuclein Phospho Ser129 because the primary antibody was biotinylated.
Validation	Validation statements are available on the following manufacturers' web-sites.<br>https://www.pel-freez.com/rabbit-tyrosine-hydroxylase-p40101-150<br>https://labchem-wako.fujifilm.com/us/product/detail/W01W0101-2648.html<br>https://vectorlabs.com/products/biotinylated-goat-anti-rabbit-igg/?srsltid=AfmBOoqxXfJ_aaRgGEuVKPiaZo_UW9Arcs3iI9ZTOr4z0MpzmYIISxQ4

Animals and other research organisms

Policy information about studies involving animals; ARRIVE guidelines recommended for reporting animal research, and Sex and Gender in Research

Laboratory animals	Male C57BL6J mice age between 14 and 17 weeks old (Jackson Laboratory), C. elegans (BY200 and UA44). We analyzed neurodegeneration of these animals at day 7 of adulthood in C. elegans.
Wild animals	The study did not involve wild animals.
Reporting on sex	Only male mice and hermaphrodite C. elegans were used in this study.
Field-collected samples	The study did not involve the samples collected in the field.
Ethics oversight	All animal protocols (2018N000086) were approved by the Massachusetts General Hospital Institutional Animal Care and Use Committee.

Note that full information on the approval of the study protocol must also be provided in the manuscript.

Plants

Seed stocks	The study did not involve plants.
Novel plant genotypes	The study did not involve plants.
Authentication	The study did not involve plants.
